# Apoptosis and Autophagy in Breast Cancer Cells following Exemestane Treatment

**DOI:** 10.1371/journal.pone.0042398

**Published:** 2012-08-13

**Authors:** Cristina Amaral, Margarida Borges, Soraia Melo, Elisiário Tavares da Silva, Georgina Correia-da-Silva, Natércia Teixeira

**Affiliations:** 1 Laboratory of Biochemistry, Department of Biological Sciences, Faculty of Pharmacy, University of Porto, Porto, Portugal; 2 Institute for Molecular and Cell Biology (IBMC), University of Porto, Porto, Portugal; 3 Department of Zoology, Faculty of Sciences and Technology, University of Coimbra, Coimbra, Portugal; 4 Center of Pharmaceutical Studies, Pharmaceutical Chemistry Laboratory, Faculty of Pharmacy, University of Coimbra, Coimbra, Portugal; H. Lee Moffitt Cancer Center & Research Institute, United States of America

## Abstract

Aromatase inhibitors (AIs), which block the conversion of androgens to estrogens, are used for hormone-dependent breast cancer treatment. Exemestane, a steroidal that belongs to the third-generation of AIs, is a mechanism-based inhibitor that binds covalently and irreversibly, inactivating and destabilizing aromatase. Since the biological effects of exemestane in breast cancer cells are not totally understood, its effects on cell viability, cell proliferation and mechanisms of cell death were studied in an ER-positive aromatase-overexpressing breast cancer cell line (MCF-7aro). The effects of 3-methyladenine (3-MA), an inhibitor of autophagy and of ZVAD-FMK, an apoptotic inhibitor, in exemestane treated cells were also investigated. Our results indicate that exemestane induces a strong inhibition in MCF-7aro cell proliferation in a dose- and time-dependent manner, promoting a significant cell cycle arrest in G_0_/G1 or in G_2_/M phases after 3 and 6 days of treatment, respectively. This was accompanied by a decrease in cell viability due to activation of cell death by apoptosis, via mitochondrial pathway and the occurrence of autophagy. Inhibition of autophagy by the autophagic inhibitor, 3-MA, resulted in a reduction of cell viability and activation of caspases. All together the results obtained suggest that exemestane induced mitochondrial-mediated apoptosis and autophagy, which act as a pro-survival process regulating breast cancer cell apoptosis.

## Introduction

Breast cancer is the most common cause of cancer death in women worldwide. Among breast cancer patients, 60% of pre-menopausal and 70–80% of post-menopausal women have hormone-dependent (estrogen receptor positive [ER^+^]) tumors [1], [2]. As estrogens play a crucial role in stimulating ER^+^ tumor growth, the suppression of their effects is considered an important therapeutic target for breast cancer treatment. Two main approaches have been successfully applied. One targets the ER directly through the use of selective estrogen receptor modulators (SERM), such as tamoxifen, or of selective estrogen receptor down-regulators (SERD), like fulvestrant. The other is achieved by the use of aromatase inhibitors (AIs) that inhibit aromatase, the enzyme responsible by the last step of estrogen synthesis, blocking the conversion of androgens to estrogens [1], [3].

Over the past three decades AIs became an effective alternative to tamoxifen, showing clinical benefits with high specificity and reduced recurrence rates [4]. The third-generation of AIs includes non-steroidal triazole derivates, anastrozole and letrozole, that act as competitive inhibitors and one steroidal derivate of androstenedione, exemestane [4], [5]. Exemestane is a mechanism-based inhibitor that is catalytically converted into chemically reactive intermediates These molecules bind covalently and irreversibly to the substrate-binding pocket of the enzyme, inactivating and producing suicide aromatase inhibition [1], [6], [7]. Wang and Chen (2006) found that exemestane destabilizes aromatase and induces its degradation by the proteosome after its irreversible inactivation [8]. On the other hand, exemestane and its principal metabolite, 17-hydroexemestane, exhibit androgenic effects as it binds with high affinity to the androgen receptor, causing in that way, lower bone loss [2], [6], [7].

The efficacy of hormonal therapy in breast cancer is based on the fact that estrogens play an important role in cancer cell survival and proliferation, essentially affecting cell cycle [9] and inducing expression of growth factors and cytokines [10], [11]. It has also been reported that estrogen deprivation causes a decrease in cell proliferation and induces apoptosis in MCF-7 cells [12], [13] and in MCF-7 xenografts [14], [15]. SERMs [13], [16], [17] and antagonists of estrogen receptor [18] induce inhibition of cell proliferation and apoptosis in breast cancer cell lines. Although recent reports showed that tamoxifen and 4-hydroxytamoxifen (4-OHT) induced autophagy [19], [20], others referred that tamoxifen treatment is associated with both types of cell death [21], [22]. It has also been reported that some AIs, like letrozole, anastrozole and formestane inhibit proliferation of breast cancer cells by inducing cell cycle arrest in G_0_/G_1_ phase and cell death by apoptosis [13], [23]. Recently, we demonstrated that the steroidal AIs 5α-androst-3-en-17-one and 3α,4α-epoxy-5α-androstan-17-one, previously synthesized in our laboratory [24], inhibit cell proliferation in various tumour cell lines [25] and induce apoptosis and autophagy in MCF-7aro cell line [26]. Nevertheless, the effects of exemestane in breast cancer cells are not totally understood. In this way, it was evaluated the biological effects of this steroidal AI in an ER-positive aromatase-overexpressing breast cancer cell line (MCF-7aro) and studied the mechanisms of cell death induced by exemestane.

## Results

### Morphological studies

**Figure 1:**
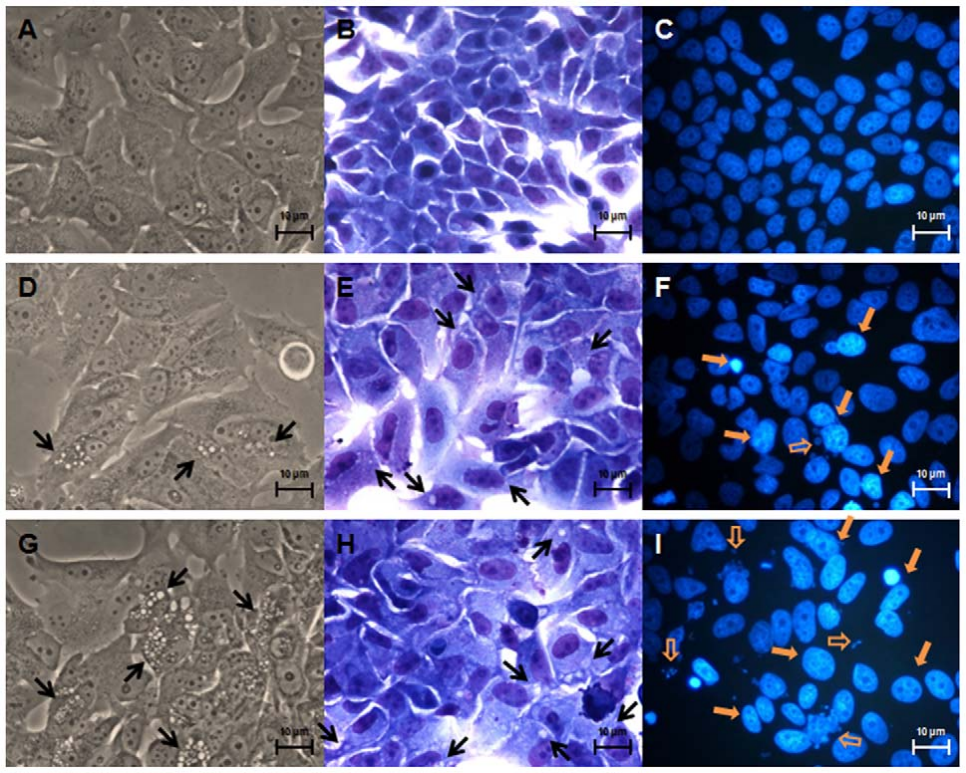
Effects of exemestane on MCF-7aro cells morphology. Phase contrast microscopy (A, D and G), Giemsa staining (B, E and H) and Hoechst staining (C, F and I). MCF-7aro cells were examined in the absence (A, B and C) or in the presence of 10 µM (D, E and F) or 15 µM (G, H and I) of exemestane during 6 days. Treated cells presented, cytoplasm vacuolization (black arrows) in phase contrast microscopy and Giemsa staining, chromatin condensation (yellow filled arrows) and chromatin fragmentation (yellow open arrows) in Hoechst staining.

To investigate the morphological alterations induced by exemestane, MCF-7aro cells, were cultured with or without exemestane during 3, 6 (Fig. 1) and 9 days and examined by phase contrast microscopy, Giemsa and Hoechst staining. After 3 days of exemestane treatment, few membrane blebbings and chromatin fragmentation were observed (data not shown). After 6 and 9 days, cells showed marked morphological alterations, like membrane blebbings, chromatin condensation and fragmentation, cytoplasm vacuolization and the presence of non-adherent cells. A decrease in cell density was also observed after 9 days of treatment (data not shown). These features were more evident for the highest concentration of exemestane and increased with the time of treatment.

### Cell viability and cell proliferation

**Figure 2:**
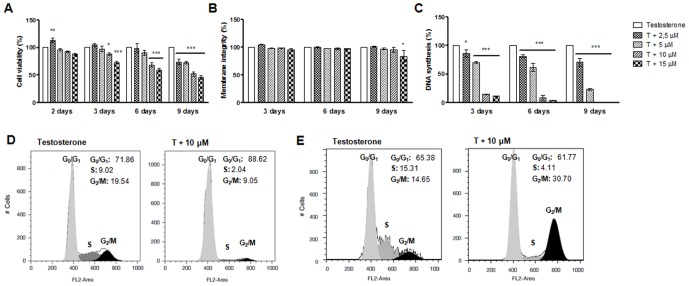
Effects of exemestane on MCF-7aro cells viability, membrane integrity, rate of DNA synthesis and on cell cycle distribution. To study cell viability (A, B) and cell proliferation (C), cells were treated with different concentrations of exemestane for different times. Cells cultured with T represent the maximum of cell viability and cell proliferation and were considered as control. To study cell cycle distribution cells were treated with exemestane during 3 (D) and 6 (E) days and subjected to flow cytometric analysis after PI staining. Data presented in histograms were analysed with FlowJo Software (Tree Star, Inc) by the application of the Watson mathematical model and are representative of one independent assay. Results are the mean ± SEM of three independent experiments, performed in triplicate. Significant differences between the control and cells with exemestane are denoted by * (p<0.05) and *** (p<0.001).

To evaluate the effects of exemestane (2.5–15 µM) in MCF-7aro cells viability and cytotoxicity, 3-(4,5-dimethylthiazol-2-yl)-2,5-diphenyltetrazolium (MTT) and lactate dehydrogenase (LDH) assays were performed for 2, 3, 6 and 9 days. After 2 days of exemestane treatment no effects in cell viability were observed. Although, after 3, 6 and 9 days and as shown in Fig. 2A, exemestane induced a reduction in cell viability that was dose- and time-dependent. The lower concentrations of exemestane (2.5–5 µM) did not affect cell viability, except in the case of 9 days of treatment. However, for the higher concentrations (10–15 µM), exemestane induced a significant decrease (p<0.05; p<0.001) in cell viability. A significant increase (p<0.05) in LDH release was only observed for exemestane at 15 µM after 9 days of treatment (Fig. 2B).

To analyse the exemestane effects in cell proliferation, thymidine incorporation assay was performed. As shown in Fig. 2C, exemestane induced a dramatic decrease in cell proliferation in a dose- and time- dependent manner. Contrary to the effect on cell viability, all concentrations and times of incubation caused a statistically significant decrease (p<0.05; p<0.001) in the rate of DNA synthesis.

### Cell cycle analysis

To identify the underlying mechanism associated with the anti-proliferative effects of exemestane, it was evaluated the effect on cell cycle progression by measuring DNA content by flow cytometry. After 3 days of treatment, different concentrations of exemestane (5–10 µM) caused a significant cell cycle arrest in G_0_/G_1_ phase and a decrease in the percentage of cells in S and G_2_/M cell cycle phases (Fig. 2D) in a dose-dependent manner. An accumulation of cells in G_0_/G_1_ phase of 75.39±1.25%, 81.94±0.25% (p<0.001) and 90.30±1.68% (p<0.001) was observed for 2.5, 5 and 10 µM, respectively, when comparing to the control (70.49±1.37%). However, after 6 days of treatment it was detected an arrest in G_2_/M phase, 20.43±1.05% (p<0.05) and 34.47±2.67% (p<0.001) for 5 and 10 µM, respectively, when comparing to the control 14.56±0.34% (Fig. 2E).

### Analysis of cell death

**Figure 3:**
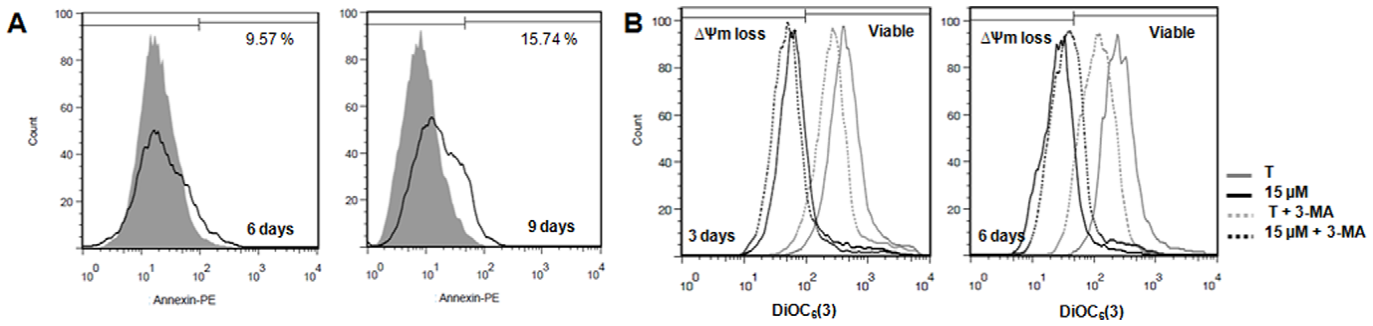
Annexin V-PE labeling and mitochondrial transmembrane potential following exemestane treatment. (A) Annexin V-PE labelling of MCF-7aro cells treated with exemestane (15 µM) for 6 and 9 days of treatment. Cells cultured with T were considered as control and for 6 days present 3.54% and for 9 days 5.58% of Annexin V binding. Data presented in histograms were analysed with FlowJo Software (Tree Star, Inc), correspond to cells gated for negative 7-AAD staining and are representative of one independent assay. The grey filled line corresponds to control and the black line to exemestane at 15 µM. (B) Mitochondrial transmembrane potential (ΔΨm) of MCF-7aro cells treated with exemestane (15 µM) with or without 3-MA (1 mM), for 3 and 6 days. Viable cells and cells with ΔΨm loss were identified. Data presented in histograms were analysed with FlowJo Software (Tree Star, Inc) and are representative of one independent assay.

To investigate the type of cell death induced by exemestane in MCF-7aro cells, assays for apoptosis and autophagy assessment were performed. For the apoptotic analysis, translocation of PS to the outer surface of plasma membrane was evaluated by Annexin V-PE binding (Fig. 3A) (Table 1). After 3 days of treatment, no differences were detected when comparing to control (data not shown). However, after 6 days, exemestane at 10 and 15 µM induced a significant increase of 2.67 (p<0.01) and 2.74 (p<0.001) times in the binding to Annexin V, respectively, compared to control. When cells were exposed for 9 days, the results were similar to 6 days, although there was a significant increase in 7-amino-acitomycin D positive (7-AAD^+^) cells (11.89%, p<0.01 for 10 µM; 17.8%, p<0.001 for 15 µM). For the concentration of 5 µM, significant differences (p<0.05) were only observed after 9 days of treatment.

**Table 1:** Effects of exemestane on Annexin V-PE labelling in MCF-7aro cells.

6 DAYS	T	T+5 µM	T+10 µM	T+15 µM	T+STS 1 µM
AnnexinV^−^/7-AAD^−^	86.69±1.62	84.45±0.15	74.47±2.53*	73.24±3.44*	59.87±1.08***
Annexin V^+^/7-AAD^−^	3.67±0.35	5.62±0.45 (1.53)	9.80±0.92** (2.67)	10.04±0.61*** (2.74)	17.40±1.15*** (4.74)
Annexin V^+^/7-AAD^+^	9.65±1.28	9.94±0.30 (1.03)	15.57±1.65 (1.61)	16.73±2.85 (1.73)	20.38±2.10** (2.11)

9 DAYS	T	T+5 µM	T+10 µM	T+15 µM	T+STS 1 µM
AnnexinV^−^/7-AAD^−^	83.83±1.22	68.98±3.42*	65.55±3.55**	60.85±2.65***	58.85±5.25***
Annexin V^+^/7-AAD^−^	5.96±0.57	11.85±1.84* (1.99)	12.57±1.07** (2.11)	15.78±1.65*** (2.65)	21.09±1.71*** (3.54)
Annexin V^+^/7-AAD^+^	9.99±1.02	19.18±2.11* (1.92)	21.88±2.85** (2.19)	27.79±0.87*** (2.78)	20.61±3.94* (2.06)

Cells were treated with different concentrations of exemestane for 6 and 9 days. Treated cells were harvested and labeled with Annexin V-PE and 7-AAD followed by flow cytometry analysis. Data are presented as viable cells (Annexin V-/7-AAD-), early apoptotic (Annexin V+/7-AAD-) and late apoptotic or necrotic cells (Annexin V+/7-AAD+). Cells cultured with T at 1 nM were considered as control and cells treated with T plus STS (1 µM) were considered as positive control for apoptosis. The data represents means ± SEM of three independent experiments done in triplicate. The ratio treatment/control is presented in bold within brackets. Significant differences between the control versus treated cells are indicated by

* (p<0.05),

** (p<0.01) and

*** (p<0.001).

To investigate the involvement of mitochondria in exemestane treatment, it was analysed the mitochondrial transmembrane potential (ΔΨm) with 3,3′-dihexyloxacarbocyanine iodide (DiOC_6_(3)) (Fig. 3B) (Table 2). After 3 days, a significant increase (p<0.001) of ΔΨm loss of approximately 12 times was observed, for 10 and 15 µM, when compared to control. After 6 days of treatment, a higher and significant (p<0.001) increase of ΔΨm loss, approximately 21 times, was detected for both concentrations. Thus, the ΔΨm loss was only time-dependent.

**Table 2:** Effects of exemestane on mitochondrial transmembrane potential (ΔΨm) in MCF-7aro cells.

3 DAYS	T	T+10 µM	T+15 µM	T+CCCP 10 µM
Viable	94,58±1,05	32,37±2,71***	28,63±2,90***	35,88±3,86***
ΔΨm loss	5,49±1,06	67,80±2,73*** (12.35)	71,59±2,90*** (13.04)	65,06±4,21*** (11.85)

	T+3-MA	T+10 µM+3-MA	T+15 µM+3-MA	
Viable	88,88±3,60	39,42±0,06***	36,89±4,42***	-
ΔΨm loss	11,23±3,63	60,80±0,14*** (5.41)	63,51±4,51*** (5.66)	-

6 DAYS	T	T+10 µM	T+15 µM	T+CCCP 10 µM
Viable	95,77±0,08	22,01±3,04***	20,75±2,07***	30,43±4,32***
ΔΨm loss	3,65±0,13	77,85±2,77*** (21.33)	79,07±2,06*** (21.66)	68,49±4,74*** (18.76)

	T+3-MA	T+10 µM+3-MA	T+15 µM+3-MA	
Viable	90,51±2,80	32,07±3,14***	28,02±1,86***	-
ΔΨm loss	8,50±2,28	67,84±3,45*** (7.98)	71,71±2,26*** (8.44)	-

Cells were treated with different concentrations of exemestane with or without 1 mM of 3-MA after 3 and 6 days. Treated cells were harvested and labeled with DiOC_6_(3) and PI followed by flow cytometry analysis. Data are presented as viable cells and cells with ΔΨm loss. Cells cultured with T at 1 nM were considered as control and cells treated with T plus CCCP (10 µM) were considered as positive control for ΔΨm loss.The data represents means ± SEM of three independent experiments done in triplicate. The ratio treatment/control is presented in bold within brackets. Significant differences between the control versus treated cells, or between the control plus 3-MA versus cells with exemestane plus 3-MA are indicated by

*** (p<0.001).

**Figure 4:**
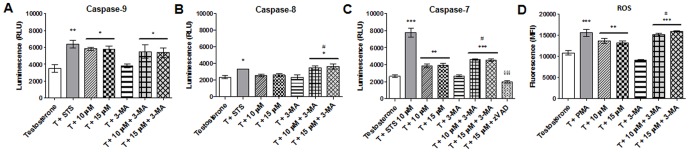
Activation of caspases and ROS production. Caspase-9 (A), caspase-8 (B) and caspase-7 (C) activities of MCF-7aro cells treated with exemestane after 3 days. The effects of 3-MA on caspase activities were also studied. Cells cultured with T were considered as control, cells treated with STS were considered as positive control and with Z-VAD-FMK was negative control. The results are presented as relative luminescence units (RLU). (D) ROS in MCF-7aro cells treated with exemestane after 3 days. It was also studied the effects of 3-MA. Cells cultured with T were considered as control and cells treated with PMA were as positive control for ROS production. The results are presented as mean fluorescence intensity (MFI). Results are the mean ± SEM of three independent experiments performed in triplicate. Significant differences between the control T versus treated cells, or between the control plus 3-MA versus cells with exemestane plus 3-MA are indicated by * (p<0.05), ** (p<0.01) and *** (p<0.001); between the cells treated with exemestane versus cells with exemestane plus 3-MA are indicated by ^#^ (p<0.05); between the cells treated with exemestane versus cells with exemestane plus Z-VAD-FMK are indicated by ^§§§^ (p<0.001).

Since mitochondrial membrane depolarization can lead to activation of caspase-9, it was evaluated caspase-9 activity by a luminescent assay. After 3 days of incubation, a significant increase (p<0.05) of approximately 67.77% (5845±191.8 relative luminescence units (RLU) and 66.23% (5793±398 RLU) was detected for 10 and 15 µM, respectively, in comparison to control (3484±459.0) (Fig. 4A). Caspase-8 activity was also determined and no significant differences were obtained after 3 days of treatment (Fig. 4B). Since there was a significant activation of caspase-9 it was also evaluated the activation of the effector caspase-7. After exemestane treatment there was a significant increase (p<0.01) of approximately 45.01% (3824±228.5 RLU) and 48.35% (3912±234.0 RLU) for 10 and 15 µM, respectively, in comparison to control (2637±161.3) (Fig. 4C).

Once the appearance of intracellular reactive oxygen species (ROS) may also be correlated with mitochondria dysfunction, it was evaluated the presence of ROS after 3 days of treatment, by the use of 2′,7′-dichlorodihydrofluorescein diacetate (DCFH_2_-DA). A significant (p<0.01) production of 25.41% (13626±624.8 MFI) and 21.52% (13203±437.91 mean fluorescence intensity (MFI)) was detected for 10 and 15 µM of exemestane, respectively, when compared to control (10865±561.54 MFI) (Fig. 4D).

**Figure 5:**
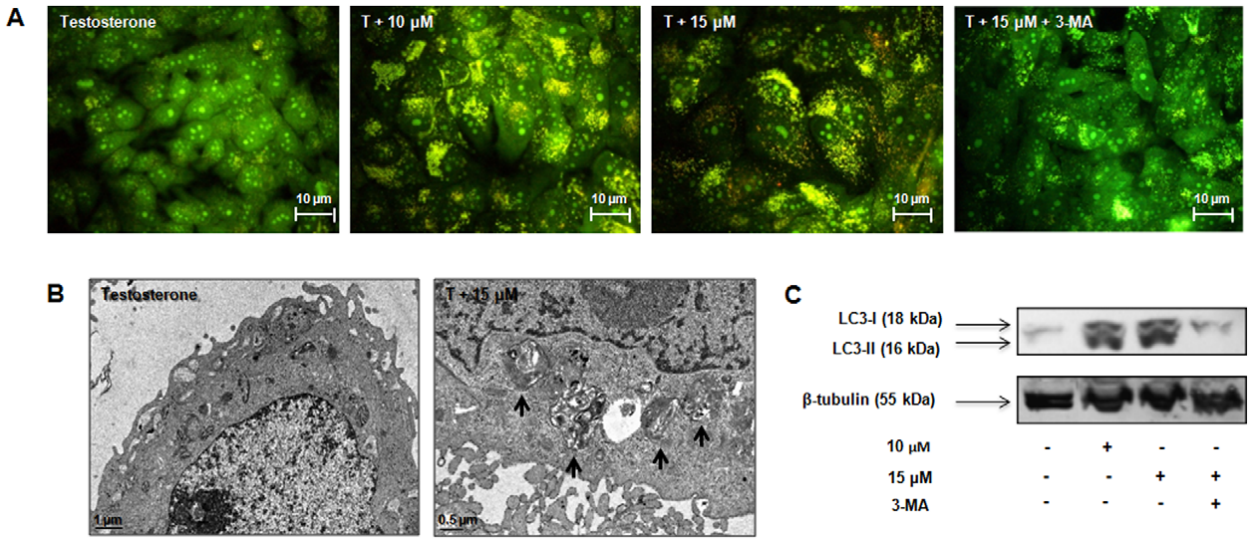
Exemestane and autophagy. (A) Effects of exemestane in the formation of AVOs analysed by fluorescence microscopy. MCF-7aro cells were treated with exemestane with or without 3-MA, during 6 days and stained with AO. The presence of AVOs was indicated by the yellow/orange/red fluorescence. (B) Ultrastructural features of cell death in MCF-7aro cells treated with exemestane for 6 days. The presence of autophagosomes (black arrows) was observed in the cytoplasm of treated cells. (C) Western Blot analysis of LC3-I/LC3-II. Cells were cultured with exemestane and with exemestane plus 3-MA for 3 days. β-tubulin was used as a loading control. Cells cultured with T were considered as control. Results are shown from a single representative of three independent experiments.

In order to clarify the nature of the cytoplasmatic vacuoles observed in contrast phase microscopy and Giemsa staining, acridine orange (AO), an acidotropic dye, was used. The lysosomotropic and pH-sensitive AO detects acid vesicular organelles (AVOs) [27], suggesting the occurrence of autophagy. AO is a cell-permeable fluorescent dye that moves freely across biological membranes and stains DNA and cytoplasm bright green. In acid compartments, such as lysosomes and autolysosomes, AO is protonated and accumulates, forming aggregates that fluorescence bright red [27], [28]. By flow cytometry analysis (Table 3), it was observed, after 3 days of treatment, an increase in AVOs formation (AO^+^) about 2 to 3.5 times (p<0.001) higher than control. Similar results were observed after 6 and 9 days. In addition, it was also detected the presence of AVOs by fluorescence microscopy. In treated cells and for all time points, it was observed alterations in green fluorescence to yellow/orange/red fluorescence that increased with exemestane concentration and time of exposure (Fig. 5A).

**Table 3:** Quantification of acid vesicular organelles (AVOs) in MCF-7aro cells treated with exemestane.

3 Days	Testosterone	T+10 µM	T+15 µM	T+H_2_O_2_
AO^−^	86.53±0.80	68.83±1.02***	52.09±1.99***	25.91±2.86***
AO^+^	13.63±0.77	31.40±1.05*** (2.30)	48.25±2.01*** (3.54)	74.65±2.97*** (5.48)

	T+zVAD	T+10 µM+zVAD	T+15 µM+zVAD	
AO^−^	78.48±3.95	51.57±4.03***	38.06±0.58***	-
AO^+^	22.17±4.10	49.88±4.48*** (2.25)	62.04±0.67*** (2.80)	-

6 Days	Testosterone	T+10 µM	T+15 µM	T+H_2_O_2_
AO^−^	78.70±1.62	40.93±2.66***	25.68±1.18***	24.62±3.74***
AO^+^	21.51±1.62	59.35±2.66*** (2.76)	74.21±1.62*** (3.45)	75.38±3.78*** (3.50)

	T+3-MA	T+10 µM+3-MA	T+15 µM+3-MA	
AO^−^	76.47±2.01	80.61±1.75***^###^	72.23±1.64***^###^	-
AO^+^	23.77±2.02	19.61±1.79***^###^ (0.82)	27.52±1.17***^###^ (1.16)	-

9 Days	Testosterone	T+10 µM	T+15 µM	T+H_2_O_2_
AO^−^	67.23±2.55	31.48±2.97***	26.11±1.24***	21.43±3.52***
AO^+^	33.20±2.64	68.80±2.96*** (2.07)	73.88±1.37*** (2.23)	82.65±3.82*** (2.49)

Cells were treated with different concentrations of exemestane after 3, 6 and 9 days. Cells with 50 µM of zVAD or with 1 mM of 3-MA, with or without exemestane (5–15 µM), were also studied for 3 and 6 days, respectively. Treated cells were harvested, stained with acridine orange and analyzed by flow cytometry. Data are presented as single cell events of viable cells, AO negative (AO^−^), and with AVO formation, AO positive (AO^+^). Cells cultured with T were considered as control and cells treated with T and H_2_O_2_ (0.05 mM) were considered as positive control for autophagic cell death. The data represents means ± SEM of three independent experiments done in triplicate. The ratio treatment/control is presented in bold within brackets. Significant differences between the control versus treated cells, or between the control plus zVAD versus cells with exemestane plus zVAD, or between the control plus 3-MA versus cells with exemestane plus 3-MA are indicated by *** (p<0.001); cells with exemestane versus exemestane plus 3-MA are indicated by ^###^ (p<0.001).

The presence of autophagosomes was confirmed by electron microscopy. After 6 days exposure to exemestane treatment (15 µM) (Fig. 5B), ultrastructural analysis revealed typical morphological features of autophagy, like giant autophagosomes in the cytoplasm, containing remnant organelles including mitochondria.

The presence of microtubule-associated protein 1 light chain 3 (LC3) protein, an autophagic marker, in cells treated with exemestane was also evaluated by immunoblotting (Fig. 5C). The autophagosome formation can be evaluated by the conversion of LC3-I to LC3-II [27]. After 3 days, exemestane-treated cells presented higher levels of LC3-II than LC3-I, confirming the presence of autophagosomes.

### Effects of autophagic and apoptotic inhibitors in MCF-7aro cells treated with exemestane

As MCF-7aro cells treated with the higher concentrations of exemestane (10 and 15 µM), presented autophagic features, like cytoplasm vacuolization and an increase of AVOs, the effects of the autophagic inhibitor 3-methyladenine (3-MA) were investigated.

When cells were treated with exemestane plus 3-MA during 3 and 6 days, it was observed a reduction in cytoplasmatic vacuolization. Moreover, the autophagic inhibitor induced a significant (p<0.01) reduction in cell viability after 3 days (88.17±2.58% to 71.67±1.25% for 10 µM and of 72.93±1.85% to 65.82±1.33% for 15 µM of exemestane without or with 3-MA, respectively) and 6 days of treatment (68.51±3.76% to 55.73±1.92% for 10 µM and of 59.28±3.79% to 46.75±2.94% for 15 µM of exemestane without or with 3-MA, respectively).

By the analysis of Annexin V-PE assay, after 6 days, any difference between exemestane with or without 3-MA (data not show) was observed, suggesting that 3-MA, in the conditions used, did not affect the translocation of PS to the outer surface of plasma membrane caused by exemestane at that time point. However, 3-MA induced a decrease in the ΔΨm loss of cells treated with exemestane (Table 2) (Fig. 3B). Comparing the ratio treatment/control of ΔΨm loss for both days, it was noted that this reduction was only dependent on time and not on exemestane concentration. In addition, in our conditions, 3-MA only induced a slight decrease of approximately 10% in the caspase-9 activity after 3 days of exemestane treatment (Fig. 4A). However, exemestane plus 3-MA induced a significant increase (p<0.05) of 37.52% (3489±235.8 RLU) and 39.62% (3615±306.3 RLU) in caspase-8 activity for 10 and 15 µM, respectively (Fig. 4B) and also a significant increase (p<0.001) in caspase-7 activity of 72.30% (4628±86.02 RLU) and 69.69% (4558±142.5 RLU) for 10 and 15 µM, respectively (Fig. 4C), when compared to control. The cells treated with exemestane plus 3-MA presented a significant increase (p<0.05) in caspase-8 and caspase-7 activity (Fig. 4B, 4C), when comparing to exemestane-treated cells.

The cells with exemestane plus 3-MA showed a significant increase of ROS of approximately 65.84% and 73.76% for 10 and 15 µM, respectively, when compared to control (p<0.001) (Fig. 4D) and to cells treated only with exemestane (p<0.05).

Inhibition of autophagy at day 6 by 3-MA almost completely abolished the AO positive staining induced by exemestane. When cells were exposed to 3-MA plus exemestane, the AVOs formation decreased to 19.61±1.79% and 27.52±1.17% versus 59.35±2.66% and 74.21±1.62% for only exemestane, respectively for 10 and 15 µM (Table 3). Statistical significant differences were also observed between cells with exemestane and cells with exemestane plus 3-MA (p<0.001). The fluorescence microscopy showed that cells treated with exemestane plus 3-MA presented less yellow/orange/red fluorescence (Fig. 5A) than exemestane-treated cells, confirming the reduction of AVOs formation.

In cells treated with exemestane during 3 days, it was detected the presence of LC3-II. In the control and treated cells with 15 µM of exemestane plus 3-MA, LC3-II was absent and only LC3-I was detected (Fig. 5C).

Since it has been referred a cross-talk between apoptosis and autophagy, it was also studied the effect of Z-VAD-FMK in AVOs formation, after 3 days of exemestane treatment. As shown in Table 3, no alteration in AVOs formation was detected. As expected, Z-VAD-FMK inhibited the activation of caspase-7 induced by exemestane (Fig. 4D).

## Discussion

This study explored the *in vitro* effects of exemestane on MCF-7aro cell proliferation, cell cycle progression and induction of cell death. This cell line is considered an important tool to study growth responses to aromatase inhibitors, as it is a breast cancer ER^+^ cell line stably transfected with the aromatase gene, that express high aromatase levels [23]. In the present work, exemestane induced a decrease in MCF-7aro cell proliferation and viability in a dose- and time-dependent manner, with no effects on cell membrane integrity, except for the higher concentration and for prolonged times of exposure. Our results revealed that the anti-proliferative effects of exemestane are essentially due to the retention in G_0_/G_1_ phase, which blocks the G_1_/S phase transition of cell cycle, preventing cells to enter in the S phase that occurs when cells are exposed to exemestane for a short period of time. However, for longer times, exemestane caused retention in G_2_/M cell cycle phase that has been referred to be associated with enhanced apoptosis and cytotoxicity [29]. Like exemestane, non-steroidal AIs, letrozole and anastrozole, also induced a decrease in cell proliferation and cell cycle arrest in MCF-7aro cells [13], [23].

In addition to inhibit cell cycle progression, exemestane caused a reduction in cell viability. This was accompanied by morphological alterations, such as membrane blebbings, chromatin condensation and fragmentation, which suggested the occurrence of cell death by apoptosis. However, the appearance of cytoplasm vacuolization, as well as the presence of AVOs also indicated the occurrence of autophagy.

To clarify the mechanism of cell death involved, it was further studied the effect of exemestane on the exposure of PS to the outer leaflet of cell membrane and on the ΔΨm. Our results revealed that exemestane induced a significant increase in the binding of annexin V and a significant increase in ΔΨm loss. In the intrinsic pathway of apoptosis, a loss of ΔΨm is associated with the release of cytochrome *c* in the cytosol and formation of apoptosome, leading to the activation of caspase-9 and of effector caspases [30]. After 3 days of treatment, cells presented a significant increase in caspase-9 activity and a significant production of intracellular ROS, suggesting the induction of apoptosis through the activation of the intrinsic pathway. However, as it has been referred that non-steroidal AIs like letrozole and anastrozole induced, respectively, a down-regulation and up-regulation of caspase-8 expression in MCF-7aro cells [23], the activity of this enzyme was also evaluated, though no significant increase was detected. The activation of the effector caspase-7 by exemestane confirms the induction of apoptosis.

In addition, by flow cytometry it was observed that exemestane induced a significant increase in AVOs with the time of exposure. Moreover, electron microscopy revealed the presence of autophagosomes engulfing cytoplasmatic fractions and organelles, such as mitochondria. During autophagy, the pro-LC3 is proteolytically converted in the cytosolic form of LC3-I, that is lipidated and translocated to autophagosome membranes to the form LC3-II, which is associated to the maturation of autophagosomes [27], [31]. The AI also induced the turnover of LC3, which is associated to the conversion of LC3-I in LC3-II. All together, our findings confirmed the existence of autophagosomes and suggest the occurrence of autophagy that seems to increase with time of treatment.

As it is referred in the literature that there is a cross-talk between these two types of cell death and some features found in apoptosis, like ΔΨm loss, chromatin condensation and PS exposure to the outer leaflet of cell membrane, may also occur in the autophagic process [32], [33], it was evaluated the effects of 3-MA in exemestane treated cells. 3-MA is an inhibitor of autophagy, which blocks the formation of autophagosomes, by controlling class I and class III phosphatidylinositol 3-kinases (PI3K) [27], [31]. As expected, the autophagic inhibitor caused a reduction in the cytoplasm vacuolization. 3-MA did neither affect the translocation of PS to the outer surface of plasma membrane nor the increase in the caspase-9 activity induced by exemestane. 3-MA did not completely abolish the drop of ΔΨm, it only reduced the mitochondrial membrane depolarization observed with exemestane, indicating that this phenomenon is due to both processes. On the other hand, cells treated with exemestane plus 3-MA presented an increase in intracellular ROS when comparing to exemestane treated cells. Thus, autophagy may be a defense mechanism against the accumulation of ROS. Autophagy may remove ROS-generating mitochondria, therefore decreasing ROS production, acting as a self-protective mechanism [34]. Moreover, the inhibition of the autophagic process induced a decrease in cell viability and an increment on caspase-8 activity, when compared to exemestane treated cells. Hou *et al.* (2011) showed that in cytoprotective autophagy, active caspase-8 is sequestered by autophagosomes and degraded by lysosomes [35]. The activity of caspase-8 controls the switch from protective to destructive role of autophagy [32]. Some reports, have demonstrate that activation of caspase-8 may be independent of death receptors activation and occur downstream of mitochondria by a mechanism not totally understood [36], [37]. The cytoprotective role of autophagy was confirmed by the significant increase in caspase-7 activity observed in cells treated with exemestane plus 3-MA. Thus, the use of the 3-MA, as shown by a dramatic reduction of AVOs and LC3 II formation, affected the machinery of autophagy, reducing the appearance of the typical features of this process and not the apoptotic markers, like Annexin V or caspase-9. However, it increased the production of ROS and the activation of caspase-8 and caspase-7 in treated cells.

On the other hand, the use of Z-VAD-FMK did not affect the autophagic process. Supporting these observations, Boya *et al.* (2005) have also demonstrated that Z-VAD-FMK did not affect the formation of autophagic vacuoles by hydroxychloroquine [38].

Different interactions between apoptosis and autophagy have been proposed. They may act as partners to induce efficient cell death in a coordinated or cooperative manner, but in this case, if one pathway of cell death is blocked the other assume or may only be activated if the other fails. Autophagy may act as an antagonist to block apoptotic cell death by promoting cell survival and stabilizing genome integrity. Autophagy may also act as enabler of apoptosis, as it does not lead to death but participates in morphologic and cellular events that occur during apoptosis that should be prevented if autophagy is inhibited [39], [40]. In our study, and in the conditions used, when apoptosis is inhibited there is no exacerbation or activation of the other mechanism. However, autophagy inhibition reduced cell viability, increased ROS production and induced activation of caspase-8 and caspase-7. Thus, it appears that autophagy induced by exemestane may act as a pro-survival process. Other authors have demonstrated that tamoxifen induced both processes of cell death and that autophagy acts as a mechanism of cell pro-survival [20], [21]. Recent works in breast cancer cell lines, also showed that autophagy induced by epirubicin [41] or sulforahane [42] acts as pro-survival mechanism, protecting cells from apoptotic cell death. Moreover, Abedin *et al.* (2007) suggested that the role of autophagy in delaying apoptosis or prolonging survival is characteristic of noninvasive breast tumor cells [43].

In cells with caspase-dependent apoptosis, autophagy can be activated by mitochondrial membrane potential loss and cytocrome *c* redistribution [44], [45], eliminating damaged mitochondria and thereby limiting ROS production and tumor cell death by apoptosis [34], [46], being together with apoptosis important for tumor suppression [47].

This is the first study that documents the biological effects and mechanisms of cell death induced by the steroidal AI exemestane, in a breast cancer cell line. The main effect of exemestane on MCF-7aro cells is on cell proliferation due to a significant cell cycle arrest in G_0_/G1 phase and G_2_/M. Moreover in the conditions used, autophagy and mitochondrial-mediated apoptosis occur simultaneously. In addition, the mitochondria have an important role in the occurrence of cell death, but autophagy may act as a pro-survival process regulating or controlling breast cancer cells from apoptosis. This study may also suggest the use of inhibitors of autophagy with exemestane in a combination therapy to sensitize breast cancer cells to death, being a promising approach for the treatment of hormone-dependent breast cancers.

## Materials and Methods

### Cell culture

The ER-positive aromatase-overexpressing human breast cancer cell line, MCF-7aro, prepared by stable transfection of MCF-7 cells with the human placental aromatase gene and Geneticin selection [48] [49] [50], was kindly provided by Dr. Shiuan Chen (Beckman Research Institute, City of Hope, Duarte, CA, U.S.A.). Cells were maintained with Eagles's minimum essential medium (MEM) supplemented with Earle's salts and 1 mmol/L sodium pyruvate, 1% penicillin-streptomycin-amphotericin B, 700 ng/ml G418 and 10% heat-inactivated fetal bovine serum (FBS) (Gibco) in 5% CO_2_ atmosphere at 37°C. To avoid the interference of steroids present in FBS and of the estrogenic effects of phenol-red [51], three days before starting the experiments, cells were cultured in an E_2_-free MEM medium without phenol-red containing 5% pre-treated charcoal heat-inactivated fetal bovine serum (CFBS). All the experiments were performed according to these conditions, with 1 nM of testosterone (T) (Sigma-Aldrich Co.), which was used as aromatase substrate and proliferation inducing agent and with or without exemestane (Sequoia Research Products Ltd.). The medium and drugs were refreshed every 3 days. Cells incubated with 1 nM of T plus 0.05% of DMSO (Sigma-Aldrich Co.) were used as control. All the assays were performed in triplicate in three independent experiments.

### Morphological studies

The morphological alterations induced by exemestane were evaluated by phase contrast microscopy, Giemsa and Hoechst staining. After treatment, cells were fixed with 4% of paraformaldehyde (Sigma-Aldrich Co.). For Hoechst staining, cells were exposed to 0.5 mg/ml Hoechst 33258 (Sigma-Aldrich Co.) for 20 min and mounted with vectashield mounting medium. The nuclear morphology was examined under a fluorescence microscope (Eclipse E400, Nikon), equipped with an excitation filter with maximum transmission at 360/400 nm, and processed by Nikon ACT-2U image software. The Giemsa (Merck) stained cells were observed under the microscope Eclipse E400, Nikon equipped with image analysis software LeicaQwin.

### Cell viability and cell proliferation

Cell viability was assessed by tetrazolium salt, 3-(4,5-dimethylthiazol-2-yl)-2,5-difenyltetrazolium (MTT) assay and by measuring the lactate dehydrogenase (LDH) release. MCF-7aro cells were cultured in 96-well plates and incubated with different concentrations of exemestane during 2, 3, 6 and 9 days. Cells were also treated with 1 mM of 3-methyladenine (3-MA, (Sigma-Aldrich Co.)), an inhibitor of autophagy. After MTT (0.5 mg/ml) (Sigma-Aldrich Co.) addition, formazan was quantified spectrophotometrically. LDH release was measured using CytoTox 96 nonradioactive cytotoxity assay kit (Promega Corporation) according to the manufacturer's protocol.

To study the effects of exemestane on DNA synthesis, ^3^H-thymidine incorporation assay was performed. At each exposure time, ^3^H-thymidine (0.5 µCi) (Amersham) was added and incubated for the last 8 hours. Cells were harvested and, after addition of scintillation cocktail, ^3^H-thymidine incorporation was determined in a scintillation counter (LS 6500, Beckman Instruments). Results are expressed as relative percentage of the untreated control cells (100%).

### Cell cycle analysis

To investigate the anti-proliferative effects of exemestane, cell cycle analysis was performed by flow cytometry. Cells were incubated with exemestane (2.5–10 µM) during 3 and 6 days, fixed with 70% cold ethanol and resuspended in 0.5 ml of DNA staining solution (5 µg/ml Propidium Iodide (PI), 0.1% Triton X-100 and 200 µg/ml DNase-free RNase A in PBS) (Sigma-Aldrich Co.) for 30 min. The three fluorescence channels (FL-1, FL-2 and FL-3) were set on a linear scale. The antiproliferative effect was indicated by the percentage of cells in G_0_/G_1_, S and G_2_/M phases of the cell cycle. Flow cytometric analysis was always based on the acquisition of 20 000 events in a Becton Dickinson FACSCalibur equipped with CELLQuest Pro software.

### Analysis of apoptosis

To evaluate the translocation of phosphatidylserine (PS) to the cell surface, Annexin V-PE apoptosis detection Kit (BD Biosciences Pharmingen) was used. Mitochondrial transmembrane potential (ΔΨm) loss was studied using 3,3-dihexyloxacarbocyanine iodide (DiOC_6_(3) (Gibco) and flow cytometry analysis. Cells were cultured in 6-well plates and treated with exemestane (5–15 µM) during 3, 6 and 9 days. Adherent and non-adherent cells, after being pooled were incubated with the corresponding dye.

Cells were stained with Annexin V-PE and 7-amino-acitomycin (7-AAD), according to the manufacturer's instructions. As positive control, cells were incubated with staurosporine (STS) (1 µM) (Sigma-Aldrich Co.) for 14 hours. Detectors for all three fluorescence channels (FL-1, FL-2 and FL-3) were set on a logarithmic scale. Bivariant analysis of Annexin-PE fluorescence (FL-2) and 7AAD fluorescence (FL-3) distinguished different cell populations, Annexin V^−^/7-AAD^−^ were designated as viable cells; Annexin V^+^/7-AAD^−^ as apoptotic and Annexin V^+^/7-AAD^+^ as late apoptotic and/or necrotic cells.

For ΔΨm, cells were treated with exemestane or with 3-MA (1 mM) with or without exemestane, during 3 and 6 days. As positive control, cells were incubated with 10 µM of the mitochondrial depolarizant agent carbonyl cyanide m-chlorophenylhydrazone (CCCP) (Sigma-Aldrich Co.) and stained with DiOC_6_(3) (10 nM) for 30 min. PI (5 µg/ml) was added prior to FACS analysis to discriminate among live cells that stain only with DiOC_6_(3), early apoptotic cells that lost the ability to accumulate DiOC_6_(3), and late apoptotic/necrotic cells that stain only with PI. Detectors were set on logarithmic scale, FL-1 was used to measure DiOC_6_(3) at green fluorescence and FL2 and FL-3 to measure PI red fluorescence.

Caspase-Glo® 9, Caspase-Glo® 8 and Caspase-Glo® 3/7 (Promega Corporation) are homogeneous luminescent assays that were used according to the manufacturer's instructions. Cells were incubated with exemestane (10–15 µM) and with 3-MA (1 mM) plus exemestane for 3 days. As positive control, cells were incubated with STS (10 µM) for 3 hours and as negative control, cells with exemestane plus Z-VAD-FMK (50 µM). The resultant luminescence was measured in a 96-well Microplate Luminometer (BioTek Instruments) and presented as relative light units (RLU). It must be noted that as MCF-7 cells are known to be caspase-3 deficient [52], the use of Caspase-Glo® 3/7 kit only evaluates the activation of caspase-7.

### Intracellular reactive oxygen species (ROS) measurement

To detect the levels of intracellular ROS the 2′,7′-dichlorodihydrofluorescein diacetate (DCFH_2_-DA) method was used. DCFH_2_-DA is a lipophilic non-fluorescent compound that crosses cell membrane and is oxidized to the fluorescent compound 2′,7′-dichlorofluorescein (DCF) [53]. Cells were incubated with exemestane (10–15 µM) and with 3-MA (1 mM) plus exemestane for 3 days. As positive control, cells were incubated with phorbol 12-myristate 13-acetate (PMA) (Sigma-Aldrich Co.) at 25 ng/ml for 2 hours. Cells were labeled with DCFH_2_-DA (50 µM) (Sigma-Aldrich Co.) for 1 hour at 37°C and fluorescence was measured using an excitation wavelength of 480 nm and an emission filter of 530 nm in a 96-well Microplate Luminometer and is presented as mean fluorescence intensity (MFI).

### Detection of acid vesicular organelles

Acridine orange (AO) (Sigma-Aldrich Co.) was used to evaluate and quantify the formation of acid vesicular organelles (AVOs), by fluorescence microscopy and flow cytometry. AO is an acidotropic fluorescent dye that stain DNA and cytoplasm bright green (AO^−^) and when protonated in the presence of acid compartments it fluorescences bright red (AO^+^). MCF-7aro cells treated with exemestane (10–15 µM) were cultured for 3, 6 and 9 days. As positive control, cells were incubated with H_2_O_2_ (0.05 mM) (Sigma-Aldrich Co.) for 14 hours. Cells with and without exemestane were also treated with Z-VAD-FMK (50 µM) (BD Biosciences Pharmingen), a pan caspase inhibitor, during 3 days or with 3-MA (1 mM) during 6 days. After incubation, cells were tripsinized and incubated with AO at 0.5 µg/ml. Green (510–530 nm) and red (>650 nm) fluorescence emission with blue (488 nm) excitation light was measured with detectors for fluorescence channels FL-1 and FL-3 set on a linear scale.

For fluorescence microscopy, cells were stained with AO at 0.1 µg/ml during 15 min. The presence of AVOs was indicated by the yellow/orange/red fluorescence, analysed in the fluorescence microscope equipped with a 490 nm band-pass blue excitation filters and a 515-nm long pass-barrier filter.

### Analysis of intracellular vacuoles by electron microscopy

Cells were cultured in 6-well plates and treated with exemestane (15 µM) during 6 days. Cells were fixed with 2% glutaraldehyde/4% paraformaldehyde (Sigma-Aldrich Co.) and post-fixed in 1% osmium tetroxide. Ultrathin sections (60 nm) were collected and stained with uranyl acetate and lead citrate, and examined using a Zeiss EM902 transmission electron microscope (Carl Zeiss Oberkochen). Images were digitally recorded using a Gatan SC 1000 ORIUS CCD camera (Warrendale).

### Western-Blot analysis

Cells treated with exemestane (10–15 µM) and with or without 3-MA were cultured during 3 days in 6-well plates. After incubation, cells were lysed with cold TNTE lysis buffer (20 mM Tris-HCl, 150 mM NaCl, 0.3% Triton X-100 and 5 mM EDTA) (Sigma-Aldrich Co.), pH 7.5 containing appropriate protease inhibitors (Sigma-Aldrich Co.), and centrifuged at 18 800×g for 5 min at 4°C. Protein concentrations were determined using a Bradford assay kit (Bio-Rad). A total of 100 µg of protein per sample were subjected to 4–20% SDS-PAGE and transferred to nitrocellulose membranes. Immunodetection was performed using rabbit polyclonal antibody anti-LC3 (1∶250) (Medical & Biological Laboratories) and the secondary peroxidase goat anti-rabbit antibody (1∶5000) (Vector Laboratories). Immunoreactive bands were visualized using a chemiluminescent substrate Super Signal West Pico (Pierce). Membranes were then stripped and incubated with rabbit monoclonal anti-β-tubulin antibody (1∶500) (Santa Cruz) to control loading variations.

### Statistical analysis

The data presented are expressed as the mean ± SEM. Statistical analysis of data was performed using analysis of variance (ANOVA) followed by Bonferroni and Dunnet post-hoc tests for multiple comparisons. Values of P<0.05 were considered as statistically significant.
